# Discrete patterns of microbiome variability across timescales in a wild rodent population

**DOI:** 10.1186/s12866-023-02824-x

**Published:** 2023-03-30

**Authors:** Jonathan Fenn, Christopher Taylor, Sarah Goertz, Klara M. Wanelik, Steve Paterson, Mike Begon, Joe Jackson, Jan Bradley

**Affiliations:** 1 School of Life Sciences, University of Nottingham, Nottingham, NG7 2RD UK; 2 University of Liverpool, Liverpool, UK; 3 University of Salford, Salford, UK

**Keywords:** Microbiome, Aging, Wild Populations, Captivity, Alpha Diversity, Beta Diversity

## Abstract

Mammalian gastrointestinal microbiomes are highly variable, both within individuals and across populations, with changes linked to time and ageing being widely reported. Discerning patterns of change in wild mammal populations can therefore prove challenging. We used high-throughput community sequencing methods to characterise the microbiome of wild field voles (*Microtus agrestis*) from faecal samples collected across 12 live-trapping field sessions, and then at cull. Changes in α- and β-diversity were modelled over three timescales. Short-term differences (following 1–2 days captivity) were analysed between capture and cull, to ascertain the degree to which the microbiome can change following a rapid change in environment. Medium-term changes were measured between successive trapping sessions (12–16 days apart), and long-term changes between the first and final capture of an individual (from 24 to 129 days). The short period between capture and cull was characterised by a marked loss of species richness, while over medium and long-term in the field, richness slightly increased. Changes across both short and long timescales indicated shifts from a Firmicutes-dominant to a Bacteroidetes-dominant microbiome. Dramatic changes following captivity indicate that changes in microbiome diversity can be rapid, following a change of environment (food sources, temperature, lighting etc.). Medium- and long-term patterns of change indicate an accrual of gut bacteria associated with ageing, with these new bacteria being predominately represented by Bacteroidetes. While the patterns of change observed are unlikely to be universal to wild mammal populations, the potential for analogous shifts across timescales should be considered whenever studying wild animal microbiomes. This is especially true if studies involve animal captivity, as there are potential ramifications both for animal health, and the validity of the data itself as a reflection of a ‘natural’ state of an animal.

**Supplementary Information:**

The online version contains supplementary material available at 10.1186/s12866-023-02824-x.

## Introduction

Gastrointestinal microbiome composition is complex and understanding the causes and consequences of changes in microbiome structure can be challenging. The microbiome can be influenced by a range of environmental factors, including infection status, nutrition, and life history, all of which can be drivers of microbiome structure and diversity [1]. Changes in the microbiome can in turn have impacts on a variety of host phenotypes [2] including infection responses, food metabolism, pathogenicity of the bacterial taxa themselves, and ultimately host fitness. While some microbial populations may be intrinsically more dynamic in abundance than others, some may also show specific shifts clearly associated with factors such as ageing [3–5], helminth infection [6, 7], and diet [8–10]. In contrast, a significant proportion of the bacterial community is comparatively stable, comprising a ‘core-microbiome’ of established taxa. The term ‘core microbiome’ is used to refer to communities that are stable either within, or between individuals (or both), and so high-resolution sampling both within and between individuals is required to expose any shifts in this core [11]. Although the important role of archaea and fungal species in microbiome communities is becoming increasingly well understood, this study focusses solely on bacterial communities, and as such ‘microbiome’ will hereafter refer to bacterial taxa.

While temporal variation around this core has been extensively examined in human populations [12–14] and laboratory model species [15, 16], the prominence and nature of consistent patterns of change across varying timescales in wild mammal populations is currently underexplored and invites further investigation. These shifts can affect both α-diversity (the local diversity of species in a community, and β-diversity (the difference in taxa between communities.) Human populations are analogous to wild animal populations, being genetically diverse, and experiencing a diversity of lifestyles, diets and immune challenges. The fact that modern humans are typically far more long-lived means ageing effects may not be as prevalent in typical wild animal populations. Despite this, age-related shifts to Bacteroidetes-dominant microbiomes have been recorded in laboratory studies of mice ZHANG, and non-model species including marmosets [17], and pigs [18]. Ageing effects may, therefore, scale somewhat to the ages of the host species, as is observed in metabolism [19], physical function [20], and mutation rates [21, 22].

Age-associated changes reported in human populations vary in nature, for instance depending on existing infections [23], but are typically characterised by increased proportions of *Bacteroidetes* species [24–26]. Such shifts may not be a linear, as the *Bacteroidetes/Firmicutes* ratio has been shown to initially decrease from infancy to adulthood, before increasing again in old age [27]. These changes are associated with dysbiosis, characterised by increased abundance of pathogenic taxa, and have been implicated or associated with poorer health [28], typically coincident with increased levels of frailty and inflammation [29], and more specific pathologies, such as kidney disease, which is associated with reduced Firmicutes and increased Fusobacteria and Proteobacteria [30]. α-diversity of gut microbiomes increases with age in humans [31], lab mice [32], and non-model species [18, 33]. Despite this, studies on some species have shown the opposite effect [17, 34]. These discrepancies may be species-specific, but may also be due to conflation of ‘chronological age’ (a simple measure of time), which is associated with increased richness and ‘biological age’ (maturation of host physiology and increasing physical frailty) which is associated with reductions in richness and associated pathology—while biological and chronological age are strongly associated, they will not always necessarily increase together in a linear fashion [3, 35].

Short-term changes, over the scale of days and hours, have been demonstrated in lab mice in response to stress [36], changes in diet [37–39], as a result of host diurnal rhythmicity [40], and in human patients following severe injury [41]. Rapid changes in faecal community structure can also occur external to the host, depending on exposure to varying environmental conditions [42]. Microbiome studies of wild populations commonly involve some element of captivity [43] (Morgan and Tromborg 2007)to allow for ease of monitoring or sample/tissue collection, and so it is important to know whether such changes are occurring following capture. Marked differences between the microbiome of laboratory and wild populations have been recorded in a range of species, with domestication or captive breeding commonly associated with reduction in microbial diversity or loss of taxa, relative to wild populations, and changes in translocated individuals occurring relatively rapidly [44–48]. Again, these shifts are not universal, with some species showing increased microbiome richness under captivity [49], and more robust microbiomes, showing reduced captivity-associated differences over time [50, 51].

Studies exploring these patterns in detail across timescales in wild populations are lacking, or are often limited. For example, a longitudinal analysis of mouse lemur microbiomes (*Microcebus rufus*) showed reduced α-diversity in older individuals, but was restricted to 15 individuals [52]. Studies of populations thatare more reliably trappable at high numbers allow us to build upon work on human, lab and wild populations, and more reliably understand the prevalence and significance of characteristic patterns of change in microbiome communities across timescales.

Microbiome studies are typically cross-sectional, allowing for invasive sampling of the gut post-cull, and thus providing what is hopefully the most accurate snapshot of the live animal’s GI microbiome community. While longitudinal microbiome studies must typically rely on faecal samples rather than direct sampling of the gut, diversity metrics have been shown to be highly correlated between faeces and caecum samples, making faeces a suitable representation of microbiome communities in the live animal [53]. Dynamic changes in the microbiome, and their environmental and host-intrinsic causes, are becoming increasingly well characterised, and so the importance of emphasising longitudinal experimental design in wild animal microbiome studies is becoming more apparent [54].

We investigated the gastrointestinal microbiome of a wild population of the field vole, *Microtus agrestis.* Using both longitudinal faecal samples from mark-recapture trapping, and faecal samples taken after capture and dissection, we examined the level of between- and within-individual variation associated with different bacterial phyla. We explored how levels of species richness, and the balance between representation of Bacteroidetes and Firmicutes as dominant phyl constitute patterns of change in microbiome structure over different timescales, as well as what ecological factors such as host condition and infection status might be associated with this variation. Patterns of changes over different timescales in wild animals are likely to be complex and somewhat population-specific, but by characterising those patterns in a well-studied population, we aim to provide an example of how such changes might manifest, and how they might be studied.

## Methods

### Trapping & dissection

Field methods are based on previous studies of this population [55, 56], and are also described elsewhere [57]. Voles were live-trapped using a grid composed of 197 Ugglan traps, over approximately one hectare of a clear-felled area in Kielder Forest, Northumbria, UK. Trapping was conducted over twelve 3-day sessions between March and August 2017, with traps checked twice each day, in the morning and evening. Traps were baited with mixed seed and chopped carrot. Newly-trapped animals were tagged with Passive Integrated Transponders (PIT Tags) on first capture to allow for re-identification. Upon first capture, animals were visually inspected, and sex and reproductive status were recorded. At each capture, snout-vent body length was measured, and total body mass was recorded. These values were used to calculate the scaled mass index (SMI), a measure of body condition [58].

For our longitudinal sampling, 428 faecal samples were collected from 206 voles. The mean number of samples per individual was 2.28, and 3.26 when excluding individuals with only one capture. In most cases these animals were then released, but at the end of each trapping session a small number of the animals were sent to the University of Nottingham for dissection, to perform gastrointestinal helminth surveys, collect caecum samples, and extract eye lenses as a proxy measure of animal age [59]. A total of 60 voles were sampled in this way, forming the cross-sectional dataset. They were housed for either one or two nights prior to dissection, fed bird seed mix and chopped carrot, and provided with water ad libitum. Animals were killed by increasing CO_2_ concentration in a sealed chamber, with death confirmed by exsanguination. Procedures were performed with approval from the University of Liverpool Animal Welfare Committee, under a UK Home Office license (PPL 40/3235 to MB. Field-to-lab workflow is shown in Fig S1.)

Morphometric measurements taken at cull include mass, snout-vent length and tail length. Eyes were removed and stored in formalin. Later, eye lenses were removed, desiccated at 60 °C for 48 h, and weighed with an electronic balance for use as an age proxy [59, 60]. Gastrointestinal tracts were removed and stored in 80% ethanol. These animals had further faecal samples taken at cull, and for 44 of those animals, caecum tissue was also taken. All faeces and caecum samples were stored at -80 °C.

## Gastrointesintal helminth surveys

Gastrointestinal tracts were dissected under dissection microscopes, and any helminth macroparasites identified morphologically and counted. Descriptions, keys or other literature on which the identifications were based are given alongside the respective helminth taxa below. Of the animals included in this study, two types of macroparasite were commonly observed – the pinworm *Syphacia nigeriana* (64.3% prevalence) (Chromadorea: Oxyuridae) [61, 62] & tapeworms (50% prevalence) (Cestoda: Hymenolepidae and Anoplocephalidae) [63]. Other species recorded in this population include *Heligmosomoides laevis* (Chromadorea: Heligmosomidae) [64]and *Trichuris arvicolae* (Enoplea: Trichuridae) [65], but were not present in the animals recorded in this study.

### DNA extraction & microbiome sequencing

DNA was extracted from faeces and caecum tissue using the DNeasy Powersoil extraction kit (Qiagen Cat. 47,016) and sent for 16S community sequencing at University of Liverpool Centre for Genomic Research. Alongside these samples, positive and negative controls were included, provided in-house at the Centre for Genomic Research, Liverpool. Primers described by Caporaso et al., 2011 [66] were used to amplify and barcode the V4 region of 16 s (detail of primers for every stage are provided in Table S1.) A total of 658 samples were submitted and 5 μl of each DNA sample at 1 ng/μl was entered into the first-round PCR with total reaction volume of 20ul, and the following conditions: 98 °C for 2 min, 20 s at 95 °C, 15 s at 65 °C, 30 s at 70 °C for 10 cycles then a 5 min extension at 72 °C. Samples were then purified with AMPure SPRI beads in a 1:1 volume ratio (Beckman Coulter, Indiana, USA), and a secondary, nested PCR was then performed to incorporate i5 & i7 Illumina adapter sequences, using the same conditions for a further 15 cycles. Samples were again purified with SPRI beads in a 1:1 volume ratio and quantified by Qubit dsDNA HS Assay (Thermo Fisher Scientific, Massachusetts, US) using the Agilent Fragment Analyzer (Agilent Technologies, Santa Clara, US). Samples which failed to amplify were not sequenced.

Final amplified libraries and controls were pooled in equimolar amounts into 8 pools according to the Qubit data, followed by size selection on a 1.5% Pippin prep gel (Sage Science Inc., Massachusetts) using a size range of 300-600 bp. Quantity of the size selected pools of amplicon libraries was completed using a Qubit dsDNA HS Assay Kit (Thermo Fisher Scientific, Massachusetts, US), while the quality and average size was assessed using the High Sensitivity DNA Kit on the Agilent Bioanalyzer (Agilent Technologies, California, US).

Subsequently, a quantitative real-time PCR (qPCR) assay, designed to specifically detect adapter sequences flanking the Illumina libraries, was performed using an Illumina KAPA Library Quantification Kit (Kapa Biosystems, Wilmington, USA). A 20 μl PCR reaction (performed in triplicate for each pooled library) was prepared on ice with 12 μl SYBR Green Master Mix and 4 μl diluted pooled DNA (1:1000 to 1:100,000 depending on the initial concentration determined by the Qubit dsDNA HS Assay Kit). PCR thermal cycling conditions consisted of initial denaturation at 95 °C for 5 min, 35 cycles of 95 °C for 30 s (denaturation) and 60 °C for 45 s (annealing and extension), melt curve analysis to 95 °C (continuous) and cooling at 37 °C (LightCycler LC48011, Roche Diagnostics Ltd, Burgess Hill, UK). Template DNA was denatured for 5 min at room temperature using freshly diluted 0.1 N-sodium hydroxide (NaOH) and the reaction was subsequently terminated by the addition of hybridization buffer. Following calculation of the molarity using qPCR data, template DNA was diluted to a loading concentration of 8 pM using the hybridization buffer. The amplicon libraries were sequenced on an Illumina HiSeq 2500 platform (Illumina Inc., California, US) with version 2 chemistry using sequencing by synthesis (SBS) technology to generate 2 × 300 bp paired-end reads. Fragmented PhiX phage genome was added to the sequence library to increase the sequence complexity.

### Bioinformatics

Base-calling and de-multiplexing of indexed reads was performed by CASAVA version 1.8.2 (Illumina) to produce 658 samples across the two runs, in FASTQ format. The raw FASTQ files were trimmed to remove Illumina adapter sequences using Cutadapt version 1.2.1 [67]. Any reads which matched the adapter sequence over at least 3 bp were trimmed off. The reads were further trimmed to remove low quality bases, using Sickle version 1.200 [68] with a minimum window quality score of 20. After trimming, reads shorter than 20 bp were removed. If both reads from a pair passed this filter, each was included in the R1 (forward reads) or R2 (reverse reads) file. If only one of a read pair passed this filter, it was included in the R0 (unpaired reads) file. To improve base quality in both read pairs, sequencing errors were corrected in both forward and reverse reads using the error-correct module within SPAdes assembler, version 3.1.0 [69] using options '–careful' and '–only-error-correction'. The average number of paired-end reads per sample was 512,850 (SD = 161,805, IQR = 145,881).

Read pairs were merged to produce a single sequence for each pair that would entirely span the amplicon using PEAR, version 0.9.10 [70]. Additionally, sequences with uncalled bases (Ns) were removed. To remove sequences originating from potential PCR primer dimers or from any spurious amplification events, a size selection was applied to select sequences between 200 and 600 bp. To remove PhiX sequences associated with indices, each sample was compared with the complete PhiX sequence (GenBank gi9626372) using BLASTN [71]. Sequences matching PhiX (E-value < 10^–5^) were filtered out of the dataset. An average of 99.67% of reads per-sample, were successfully aligned, which was reduced to 98.57% post PhiX-filtering.

Sequences passing the filters for each sample were concatenated into a single file, which was used subject to a custom analysis pipeline based on QIIME 1.9.1 [66]. The RDP classifier was used against the GreenGenes database (version 13.8).throughout the analysis. To identify sequence variability in each sample, amplicon sequences and assigned to clusters according to sequence similarity, using SWARM version 2.2.1, [72]. To calculate the abundance of each cluster, sequences were aligned on the identified centroid clusters sequences, using a minimum similarity threshold of 97% for the entire length of the sequence.

Taxonomic assignment of each cluster (now referred to as operational taxonomic unit, OTU) was carried out using the QIIME script ‘assign_taxonomy.py’, using the RDP classifier [73] to match the centroid sequence of each cluster obtained by swarm, to a sequence from the database. The abundance table was post-processed to remove any OTU below 0.005% of the total sequence count of sequences [74].

### Statistical analysis

All statistical analyses were carried out in R 3.6.2 [75], and read counts were centered log-ratio (CLR) transformed using the ‘SleuthALR’ package [76]. The package ‘phyloseq’ was used to calculate measures of α-diversity. Three metrics were chosen to assess different aspects of α-diversity, with Shannon index values emphasising taxa evenness, phylogenetic diversity (hereafter, ‘PD’) being correlated with evenness [77] and Chao1 being suitable for datasets skewed to low-abundance taxa [78] Bray–Curtis and weighted UniFrac (wUniFrac) distances were calculated with phyloseqand used in Non-Metric Multidimensional Scaling (NMDS) to provide individual (site) scores (wUniFrac K = 5, stress = 0.0012, Bray–Curtis K = 3, stress = 0.164. In addition, robust principal component analysis (RPCA) was performed using the ‘rospca’ package [79]. UniFrac distance incorporates OTU relatedness data from a provided phylogenetic tree, and wUniFrac adjusts this distance to reduce the influence of rare OTUs and alleviate any oversized influence of rare taxa by taking abundances into account. Bray–Curtis is an abundance-based metric, whereby distance values give a measure of between-sample dissimilarity, but which are sensitive to presence of rare taxa. RPCA is also abundance-based, but can better deal with sparse, highly-dimensional datasets. Three OTUs of a total 1321 were excluded due to severe overrepresentation in specific individuals, and causing issues with MDS and RPCA ordination. For both α- and β-diversity measures, multiple indices were calculated to provide a more comprehensive picture of microbial diversity, and capture any variation more weighted to specific diversity metrics.

Using CLR abundance data, the coefficient of variance (CoV) was calculated for each OTU, based on within-individual variation from longitudinal faecal samples, and cross-sectionally between individuals at cull. These values were normalised using Box-Cox transformation, a power-transformation for positive non-normal data (Longitudinal λ = 0.51, Cross-Sectional Lambda λ = -0.42) in R [75] using the ‘car’ and ‘MASS’ packages [80, 81]. Transformed data was then used to measure correlation between variation in OTUs from cross-sectional faecal samples and longitudinal faecal samples, to ascertain bywhether OTUs which were variable between individuals were the same as those which were variable within the same individuals over time. Average OTU variance per phylum was compared by linear model, with post-hoc Tukey analysis to determine differences in variability between major phyla (phyla with fewer than 10 OTUs present, and unclassified OTUs, were excluded).

Both cross-sectional faecal and longitudinal faecal community datasets were subject to variance partitioning using the ‘VariancePartition’ R package to ascertain the ecological factors associated with variation in CLR abundance [82]. These factors include age category (designated as ‘mature’ or ‘juvenile’ from physical inspection of body size), the within-year Julian date (i.e. day number 1–365), sex and scaled mass index (SMI) [58] as a measure of condition. Julian date was chosen to account for seasonal affects as it offers a more precise continuous measure of time than simply including the trapping session, and as all samples in this analysis were collected in 2017, there was no need to account for inter-annual patterns of variation. For cross-sectional cull samples, the days kept in captivity and prevalence of gastrointestinal helminth infections were included, and for longitudinal samples, the individual ID.

Subsequent analyses were carried out to determine changes in α- and β-diversity over three time-scales; short-term changes from field to lab following capture, medium-term changes between sequential trapping sessions, and long-term changes between the first and last longitudinal samples of individual voles. All changes were assessed in R through Gaussian linear mixed models (LMER) using the *lme4* package [83] and tested for significance with ‘lmerTest’ package [84], with individual ID as a random effect, and incorporating the Julian date from the start of the year as a fixed effect to account for any seasonal changes. These models were performed on α-diversity metrics, including total unique OTU count as a measure of richness, and Bray–Curtis, wUniFracand RPCA scores to measure changes in β-diversity.

Short-term models compared samples taken at cull with the most recent longitudinal sample from the same animal (1–2 days prior). Medium-term models incorporated all consecutive longitudinal faecal samples with no missed trapping sessions between sessions (12–16 days between each sample), and assessed the size of changes observed from the first capture in the sequence as explained by the number of sessions passed. Long-term models compared paired longitudinal samples from the first and last captures, excluding all cases where that time difference was only one trapping session, with time between first and last samples also included as a factor. Trapping date was included as a factor in all models, in an effort to show changes in diversity, regardless of the time of year in which the trapping sequence occurred. However, seasonal effects can still not be completely discounted, as all traps were recorded within 2017, and as such, a general seasonal change in microbiome diversity affecting all animals from early Spring to Autumn would be difficult to distinguish from any effects due purely to ageing.

In order to assess associations between diversity metrics and other ecological and environmental factors, linear models were performed on measures of cull faecal α-diversity and Bray–Curtis and wUniFrac site scores to measure changes in β-diversity, which incorporated Julian date, scaled mass index (SMI) as a measure of condition, sex and prevalence of two gastrointestinal helminths which are common in the population – the pinworm *Syphacia obvelata* and tapeworms (Class: Cestoda). In order to incorporate infection data from gut dissections, these analyses were performed on a reduced cross-sectional dataset, using only cull faecal data.

## Results

### Taxonomic structure & variance

**Fig. 1:**
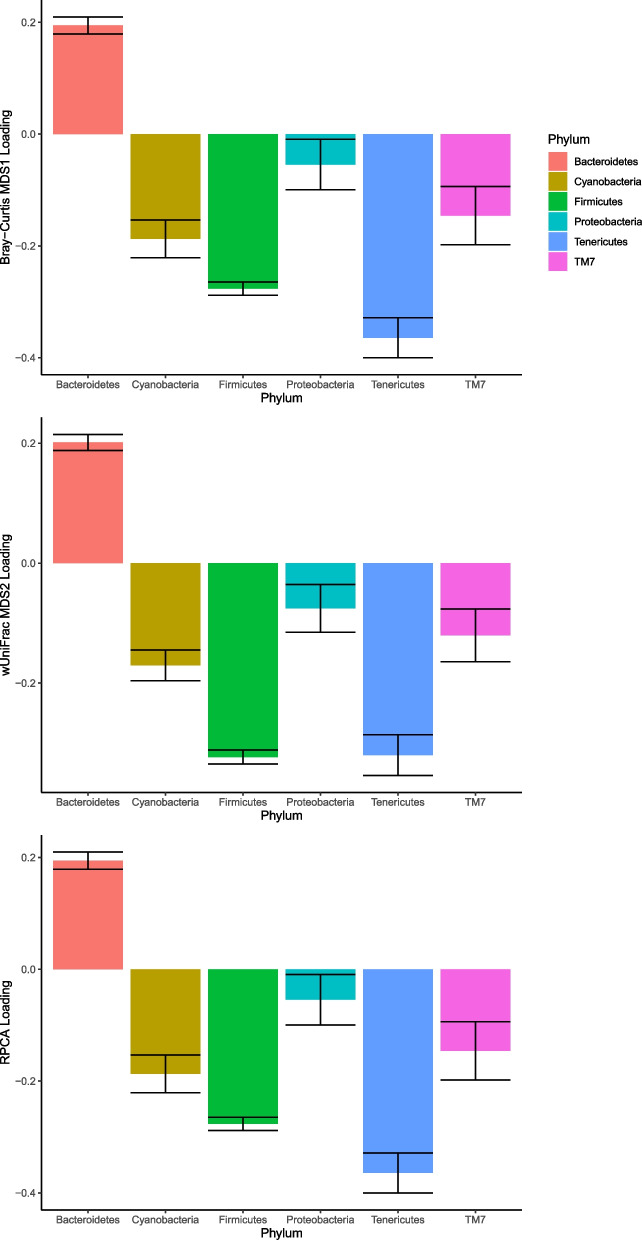
Bar chart showing inter-phylum differences represented in loadings of Bray–Curtis MDS1, Weighted UniFrac MDS2 and RPC1, distinguishing centered log-ratio abundances of Bacteroidetes taxa relative to other phyla. Error bars show standard error values

Across all sample samples, Firmicutes and Bacteroidetes constituted the majority of OTUs sequenced, (summarised in Figs. 4 and 6). Bray–Curtis MDS1, wUniFrac MDS2 and RPC1 allprovided an axis which clearly distinguished relative prevalence of these two phyla, with higher scores of both Bray–Curtis MDS1 and wUniFrac MDS2 and RPC1 representing a significant shift towards a more Bacteroidetes-dominant microbiome (Fig. 1). (OTUs most strongly represented in loadings are in Tables S3, S4 & S5).

**Fig. 2:**
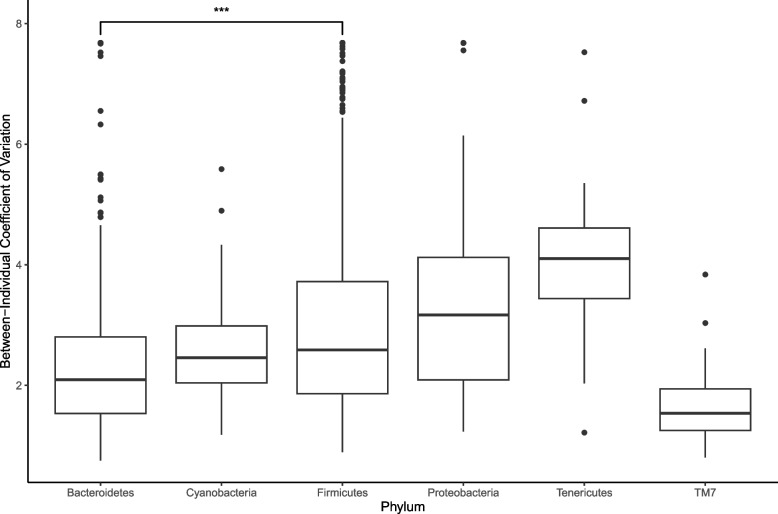
Differences across phyla in coefficient of variance in OTU CLR abundance, as sampled from cross-sectional faecal samples. Boxes represent interquartile range (IQR) with median line. Upper and lower whiskers correspond to highest and lowest values at no more than 1.5 × IQR. Individual points represent outlier OTUs. Firmicutes show a higher level of variance than the other dominant phylum Bacteroidetes (*p < 0.05, ** *p* < 0.01, *** *p* < 0.001)

Within-individual OTU variance, measured from longitudinal faecal samples, was consistent with between-individual variance of OTUs from cull faecal samples, indicating that bacterial taxa which are more variable between individuals are also more variable within an individual over time (Linear model, *p* =  < 2.6 × 10^–16^, Adjusted R^2^ = 0.78; see Fig S2). OTUs of phylum Firmicutes were significantly more variable than those of Bacteroidetes (GLM with Tukey post-hoc: within-individual *p* < 0.0001, faeces *p* < 0.0001) (Cross-sectional differences shown in Fig. 2).

### Variance partitioning

**Fig. 3:**
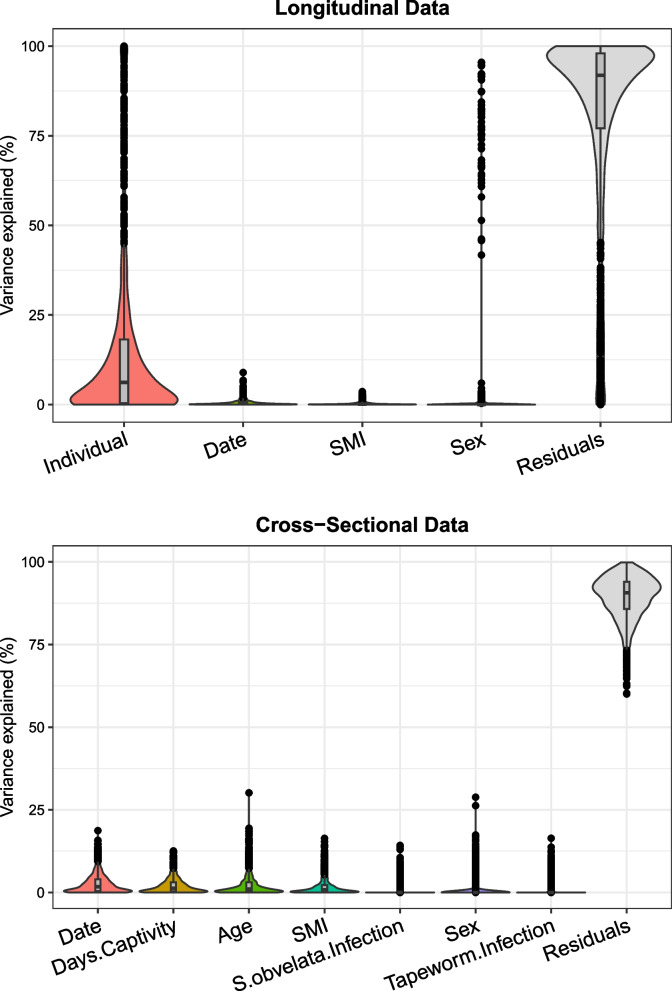
Variance partitioning of OTU CLR abundance from faecal samples. Violin plots are composed according to the percentage of variation in the CLR abundance of each OTU explained by the corresponding factor listed on the x-axis. Variation which is unexplained by the provided parameters is shown in the ‘Residuals’ violin on the right-hand side of the plot. Variance partitioning is shown for longitudinal and cull samples in the top and bottom plots, respectively

Variance partitioning was performed to show the percentage of variation in theCLR abundance of each OTU that is explained by each variable used in statistical models (Fig. 3). For the longitudinal data, these variables include individual ID, the Julian date, body condition (SMI), while for the cross-sectional data they include Julian date, the days spent in captivity prior to cull, SMI, eye lens mass as a proxy for age, sex, and prevalence of pinworm (*Syphacia obvelata*) and tapeworm infections. In the longitudinal data individual ID typically explained 8–25% of variation in most OTUs, indicating a substantial level of within-individual consistency in microbiome structure. A small number of OTUs were strongly associated with sex – of 46 OTUs which had over 50% of their variation explained by sex in the longitudinal dataset, over half [29] were of order Clostridiales. Variance partitioning of cross-sectional data found that ecological factors explained a small amount of variation for most OTUs, with 3 OTUs having over 50% of their variation explained (2 of order Bacteroidales, 1 of phylum Tenericutes). There is, however, still a great deal of unexplained residual variation across both datasets for the majority of OTUs.

### Short-term changes (Field to Lab)

**Fig. 4:**
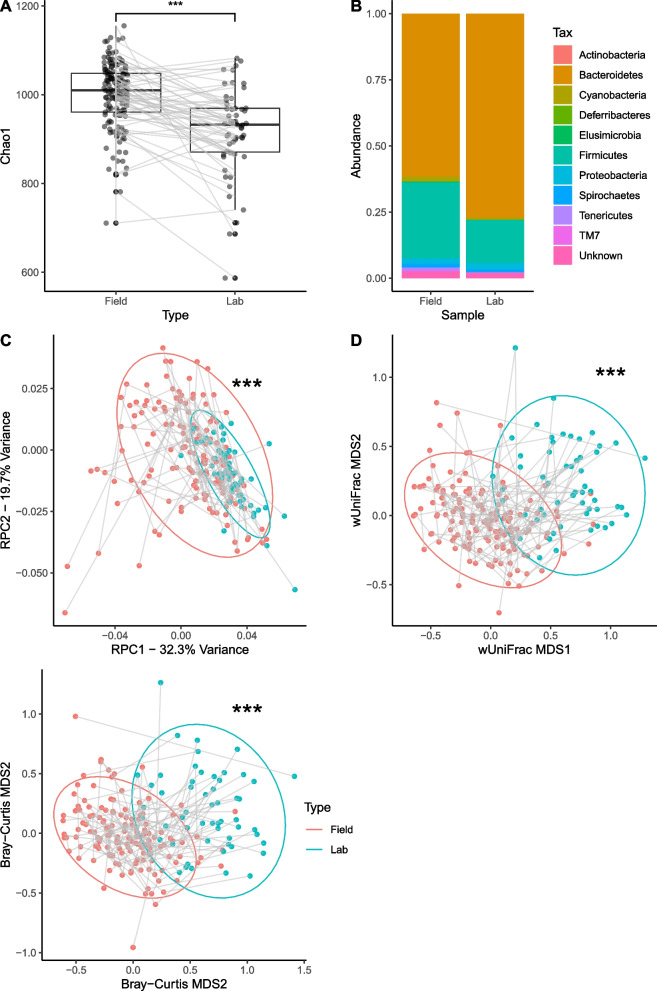
**A** Within-individual changes in α-diversity observed between paired faecal samples taken at cull, and the most recent longitudinal sample (1–2 days prior), showing a decrease in α-diversity (Chao1 index). Boxes represent interquartile range (IQR) with median line. Upper and lower whiskers correspond to highest and lowest values at no more than 1.5 × IQR. **B** By-phylum differences in mean CLR abundances of OTUs between cull faecal samples and faecal samples taken from live-captures preceding cull. Biplots showing within-individual changes in β-diversity observed between paired faecal samples taken at cull, and the most recent longitudinal sample (1–2 days prior) in **C** RPCA, **D** wUniFrac and **E** Bray–Curtis distances. Significance values are reported on RPC1, wUniFrac MDS2 and Bray–Curtis MDS1 (*p < 0.05, ** *p* < 0.01, *** *p* < 0.001)

(Summary of model outputs is provided in Table S2). Shifts in microbial α-diversity were recorded between faecal samples from the field and matched samples from the lab 1–2 days later. Faecal samples taken at cull showed significant decreases in α-diversity compared to paired longitudinal samples (LMER Chao1 p = 1.60 × 10^–8^, PD *p* = 2.35 × 10^–12^, Shannon *p* = 6.29 × 10^–15^). Significant differences inBray-Curtis (GLMER *p* < 2 × 10^–16^),wUniFrac (GLMER *p* = 2.33 × 10^–7^) and RPCA (GLMER *p* = 6.12 × 10^–8^) indicate shifts in β-diversity corresponding to a more uniform, Bacteroidetes-dominant microbiome upon arrival in captivity. Observed short-term changes are summarised in Fig. 4.

### Medium-term changes (between successive trapping sessions)

**Fig. 5:**
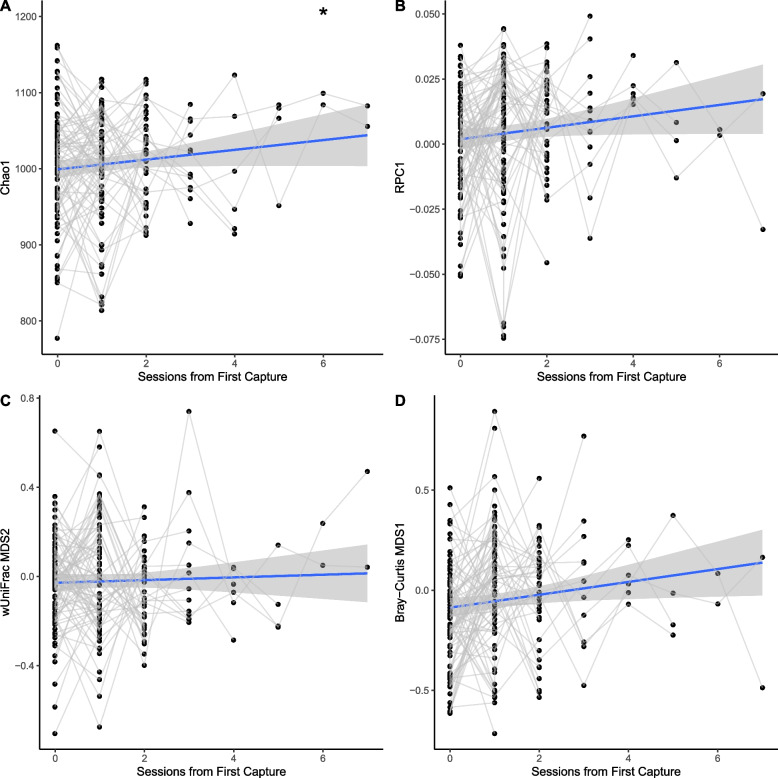
**A** Within-individual changes in α-diversity observed across sequential trapping sessions, showing increases in Chao1 α-diversity over subsequent captures from the first in the continuous sequence. **B** Within-individual changes in β-diversity observed across sequential trapping sessions, showing increases in site scores of **B** RPCA, **C** wUniFrac and  **D** Bray-Curtis distances over time, are non-significant (lines represent fitted linear model, with shaded area representing 95% confidence intervals, *p < 0.05, ** *p* < 0.01, *** *p* < 0.001)

Microbiomes showed increases in measures of α-diversity between successive trapping sessions, though only in Chao1(LMER, Chao1 *p* = 0.0177, PD = 0.279, Shannon *p* = 0.97). The inclusion of individual ID as a random effect means this is independent of any population level effects such as survival bias, and less likely to be due to seasonal effects, as any observed shifts are relative to each animal’s starting state, regardless of how it is being influenced by environmental variables at that time. While all three measures of β-diversity did show slight shifts towards Bacteroidetes dominance between sessions, these changes were not statistically significant. Observed medium-term changes are summarised in Fig. 5.

### Long-term changes

**Fig. 6:**
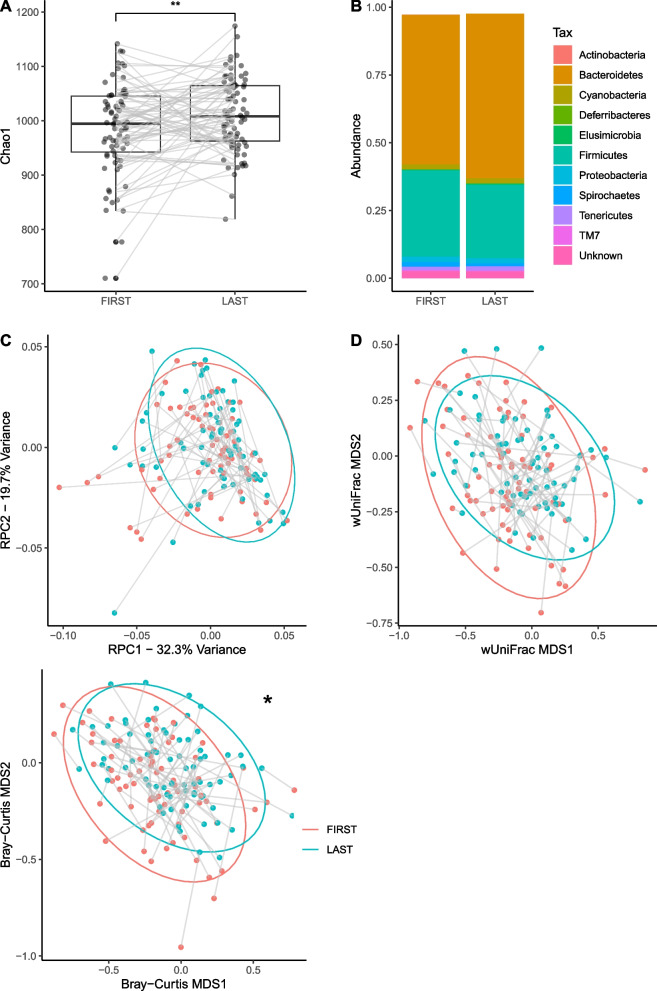
**A** Within-individual changes in α-diversity observed between paired first and last longitudinal faecal samples, showing an increase in α-diversity (Chao1 index). Boxes represent interquartile range (IQR) with median line. Upper and lower whiskers correspond to highest and lowest values at no more than 1.5 × IQR. Individual points represent outliers. **B** By-phylum differences in mean CLR abundances of OTUs between first and last live-capture faecal samples. **C** Biplot showing within-individual changes in β-diversity observed between paired first and last longitudinal faecal samples by site scores on the first two axes of variation observed in **C** RPCA, **D** wUniFrac, and** E** Bray–Curtis distances. Significant associations are found on axis 1 of Bray–Curtis ordination.. (*p < 0.05, ** *p* < 0.01, *** *p* < 0.001)

α-diversity was significantly higher at the final longitudinal capture relative to the first, indicating an accrual of bacterial species over long periods (LMER, *p* = 0.0035, PD *p* = 7.97 × 10^–4^, Shannon *p* = 0.0248). A significant difference is observed in Bray–Curtis distance between first and last captures (GLMER, *p* = 0.043), indicating a shift towards a more Bacteroidetes-dominant microbiome. No significant difference was observed in wUniFrac RPCA distances (GLMER: wUniFrac *p* = 0.058, RPCA *p* = 0.149). Observed long-term changes are summarised in Fig. 6.

### Ecological associations

Cull faeces α-diversity was positively associated with body condition, measured as SMI, though not in Shannon diversity (GLM, Chao1 *p* = 0.00323, PD *p* = 0.00861, Shannon *p* = 0.51884), indicating that richer microbiome communities are associated with better host health. SMI was also significantly associated with the Bray–Curtis distance, with Bacteroidetes dominance associated with lower condition (GLM, *p* = 0.0126), but not wUniFrac (GLM *p* = 0.162) or RPCA (GLM *p* = 0.798) distance. Tapeworm infection was positively associated with Bacteroidetes dominance as explained by Bray–Curtis (GLM, *p* = 0.0313), and RPCA (GLM, *p* = 0.00931) distances, but not wUniFrac (GLM, *p* = 0.162) (These associations are summarised in Fig S4).

## Discussion

Differences between bacterial taxa in how they are affected by temporal patterns are a crucial element of wild animal ecology, as taxon-specific shifts in microbiome structure have been implicated in many aspects of host health [85]. Here we have shown clear and defined patterns of change in gastrointestinal microbial diversity across different time scales, including long-term ageing-associated increases in α-diversity and shifts in β-diversity towards Bacteroidetes-dominance, and rapid reductions in α-diversity associated with capture and captivity. Firmicutes taxa showed significantly higher levels of variation than those of Bacteroidetes, and so while increases in α-diversity suggest an accrual of bacteria over time, it is likely that shifts in β-diversity are at least in part explained by loss of Firmicutes species. These phylum-level differences in stability have been reported in human populations, presumably due to differences in susceptibility to changes in environmental conditions [86]. These observed changes increase our understanding of natural variation in microbiome structures in wild populations, show clear parallels to analogous health-related shifts observed in ageing humans, and provide important context to consider when performing any analysis of wild animals involving captivity or rehousing. Increases in α-diversity with age, over both medium-term and long-term timescales show a pattern of constant species accrual throughout an animal’s lifetime, while positive associations with species richness and body condition suggest an important role in this process for maintaining host health.

The underlying taxonomic composition was broadly what would be expected of mammalian gastrointestinal microbiomes [87, 88], and has been recorded in other wild rodent populations [89] with Firmicutes and Bacteroidetes being the dominant phyla. While Bacteroidetes was generally more prevalent than Firmicutes in this population, in domesticated populations of *Microtus ochrogaster* Firmicutes was the more dominant phylum [90]. Most variation in OTU abundance was either associated with the specific animal, or was unexplained by any of the accompanying data. This unexplained variation may arise from purely stochastic processes, and/or there may be factors not captured in this study which play a role in shaping microbiome communities, such as dietary variation [89]. While field voles are predominantly herbivorous, variation within the plant species consumed, or occasional consumption of other foods like insect larvae may influence microbiome composition [91–93], and so alteration in diet after capture may have an impact on microbiome communities**.** Recent research in wild rodent population has also highlighted a potentially significant role for social network structure in determining population-level microbiome diversity [94]. While host-genetic influences not accounted for in this study, the impact of the genome on microbiome structure has shown to be secondary to environmental and temporal factors in both humans [1] and rodents [95].

While we have characterised distinct patterns of variability across different timescales, it should be noted that the observed short-term changes are likely to have limited relevance to ecological processes that would occur without human intervention, as the conditions and circumstances of animal capture and rehousing are not directly analogous to any naturally occurring process. Despite this, they do still illustrate that dramatic shifts in microbiome structure *can* occur in short timescales dependent on surrounding conditions, and that any microbiome study involving captivity and capture may be significantly confounded, even when efforts are made to minimise interference. With these caveats, we have been able to identify specific and directed changes in the microbiome across multiple time scales.

### Short-term changes and effects of capture

α-diversity was reduced between paired final live-capture and cull samples, across multiple richness indices, indicating rapid diversity loss associated with capture and 1–2 days of captivity. Reduced α-diversity associated with captivity has also been reported in a number of mammals [96], though this effect is not universal, and in some instance the opposite may be observed [97, 98]. The changes observed in this study both confirm that captivity-associated changes in microbiome are common across species and populations, and show how rapidly these changes can arise. There are many potential direct causes of these changes; changes in diet have been suggested as a key contributor [38, 96], but other factors such as stress [99, 100], disruption to diurnal sleep cycles [101], or combinations of these factors [102] may also play a role. These changes confirm that the microbiome can be highly volatile, and captivity-associated changes are essential to consider, both in terms of health of captive animals, and for studies of wild populations. This is particularly relevant for studies involving live trapping and rehousing of animals prior to sample collection, as changes from ‘natural’ conditions could significantly impact the microbiome of the animal in question within a short window of time. It is unlikely that these changes will be universal in nature or magnitude across study species, as captivity has been shown to have differing impacts on the gut microbiome of different host species, even within the same genus, potentially having a reduced impact on generalists compared to specialists [103, 104].

A significant taxonomic shift in microbiome communities was also observed following capture. While such shifts are in the same direction as what would be expected from ageing (increased Bacteroidetes-dominance), the magnitude and speed of the change suggests that capture and captivity itself is impacting the microbiome. Alongside the decreases in species, this indicates a loss of Firmicutes, resulting in increased prominence of Bacteroidetes. Shifts towards Bacteroidetes-dominant microbiome following captivity have been observed in deer mice *(Peromyscus maniculatus)* examined pre- and post-captivity [48], as well as in comparisons between captive mammals and counterpart wild populations [96, 97, 105, 106].

### Medium- & long-term changes in microbiome structure

Both the medium-term (between successive trapping sessions) and long-term (between first and last live-traps) models showed that microbiome α-diversity increases with age, regardless of the time of year, with no obvious drop-off observed in the oldest voles. They also showed that β-diversity shifted toward a more Bacteroidetes-dominant microbiome throughout an animal’s life. Due to the diversity metrics used in this study, some of these patterns may be driven in part by rare taxa having an outsized impact. One Bacteroidetes OTU in particular has a very strong representation in Bray–Curtis MDS1, but was not classified below phylum level (Table S 3). OTUs which strongly contributed to Firmicutes dominance in Bray–Curtis and RPCA distances were of the genus *Ruminococcus*, a taxon which is important in cellulolytic metabolism, and so loss of these bacteria over time may have an impact on host digestion and health [107] Age-associated increases in α-diversity are recorded in humans, from infancy to adulthood [26]. Age-related shifts in taxonomic structure of the microbiome have been recorded in laboratory mice [108] and captive mammals [17, 109], with multiple human studies showing specific age-related shifts to Bacteroidetes [26, 27]. The combination of changes in both α- and β-diversity observed in this study suggest that as the voles age, they accrue new bacterial OTUs, primarily of Phylum Bacteroidetes.

### Other correlates of microbiome diversity and fitness implications

While most ecological factors were not associated with microbiome diversity, body condition, measured as SMI, was found to be positively associated with both α-diversity, and a more Firmicutes-dominant microbiome. The association with α-diversity may suggest that while animals in different life stages and under different constraints may harbour qualitatively different microbiome communities, it is the richness of those communities which is most significant for host condition. The positive impacts of age and infection on α-diversity highlight how the state of the gut can be associated with multiple ecological factors which may be having indirect effects on host condition via the microbiome. On the other hand, rapid reductions in α-diversity indices associated with capture and captivity are essential to consider as context for microbiome analyses of wild and captive populations, and when considering questions of captive animal health and welfare. The negative association between Bacteroidetes and scaled mass index mirrors what has been observed in human populations, although in humans, high-BMI, particularly in obese individual, is associated with Firmicutes-dominance, and thus higher mass indices broadly corresponded to pathology, rather than healthy condition [110, 111].

The Bacteroidetes/Firmicutes ratio is commonly implicated as a key factor affecting gastrointestinal, and general, health, and so this change may be relevant to health and survival over time in wild populations. Many studies have looked at the Bacteroidetes/Firmicutes ratio as an important correlate of gut health, with alterations in this ratio linked to obesity and other pathologies [112, 113]. How these associations relate to ageing-related shifts in microbiome, in which both richness and Bacteroidetes-dominance increase is unclear, and this element of the analysis is somewhat limited by the reduced cross-sectional dataset. Further investigation could elucidate whether microbiome-associated variation in condition is primarily the result of inter-individual differences, or could be relevant to within-individual changes throughout an animal’s life.

## Conclusion

The gastrointestinal microbiome is complex and dynamic, particularly in wild, heterogeneous populations, and the factors underlying temporal changes in its composition are often difficult to determine. Using both longitudinal and cross-sectional data from a well-characterised wild rodent population, we have established robust temporal patterns of changes in microbiome structure, both in short periods following change in environmental conditions, and over the course of an animal’s life in the wild.

While microbiome structure was found to be highly individual, broad patterns of change over different timescales can be observed in both α- and β-diversity. Ageing is associated with slight shifts towards Bacteroidetes-dominance and increase in species richness, while captivity was associated with marked and larger short-term increases in Bacteroidetes-dominance and drops in species richness. Firmicutes OTUs, being significantly more variable than those of Bacteroidetes, are likely responsible for many of the temporal patterns of change in microbiome structure which were observed.

These results provide a useful framework with which to understand time-sensitive, consistent changes in microbiome structure within wild animal populations. The specific characteristics of changes observed in this population invite direct comparisons with analogous findings from laboratory rodent models and human populations, and suggest a potentially significant role for temporal microbiome dynamics on host health and fitness.

## Supplementary Information

**Additional file** **1.**

## Data Availability

Full read count data metadata, taxonomy data and phylogenetic tree are publicly available on the Dryad repository: https://doi.org/10.5061/dryad.08kprr559.
