# Supplementary material for: Discrete patterns of microbiome variability across timescales in a wild rodent population
Source: BMC Microbiol. 2023 Mar 30;23:87. doi: 10.1186/s12866-023-02824-x (PMC10061908; doi:10.1186/s12866-023-02824-x)

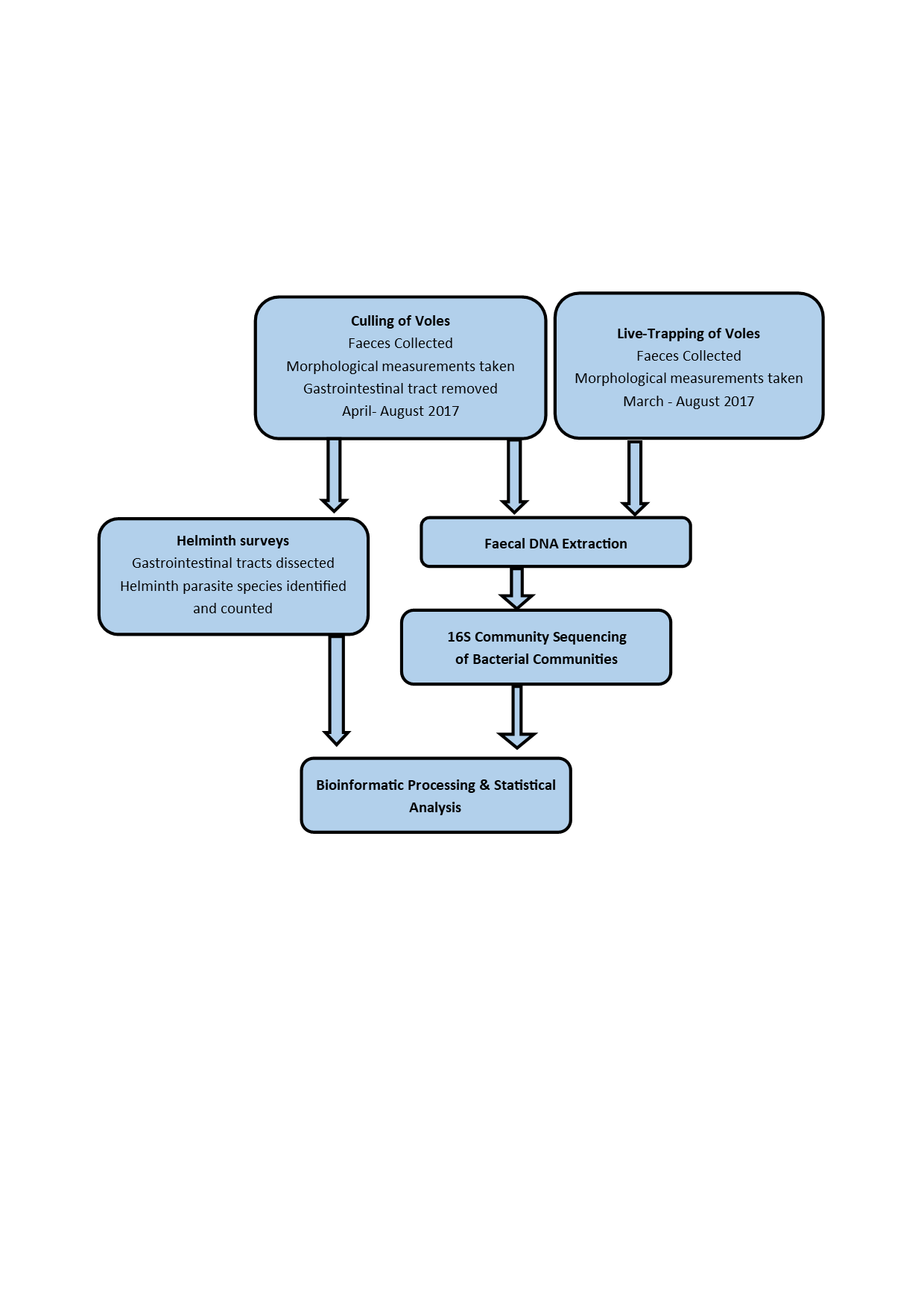


Fig S1. Overview of workflow plan from live-field trapping of voles through to cull & dissection, and downstream laboratory and molecular work.


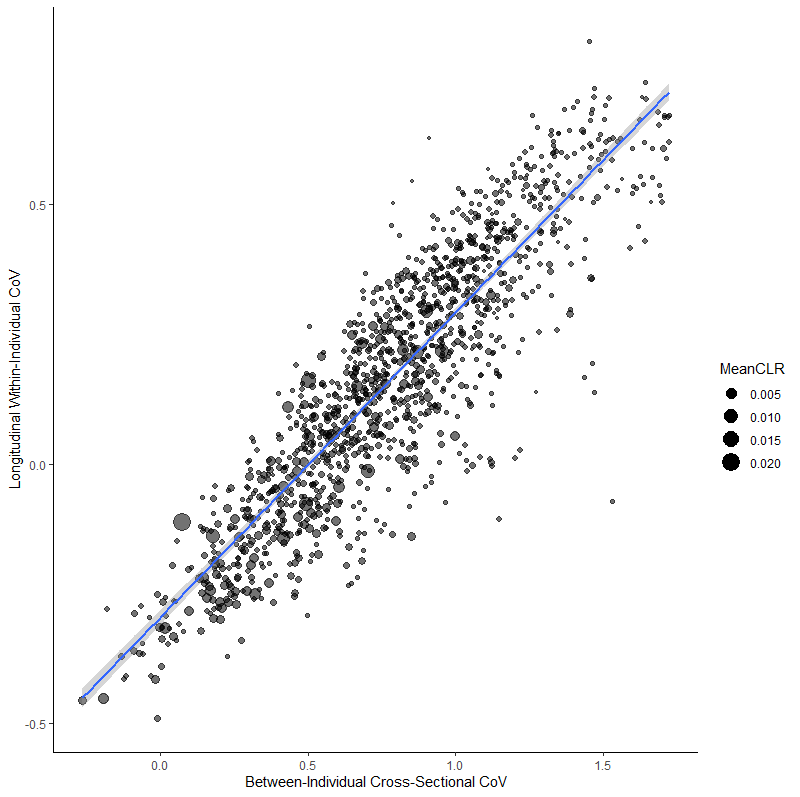


Fig S2. Positive association between OTU coefficient of variation (Box-Cox transformed) between within-individual longitudinal and cross-sectional faecal samples. Line represents linear relationship between measures, with surrounding shaded area corresponding to 95% confidence intervals (*p<0.05, ** p<0.01, *** p<0.001). Point size is proportional to average OTU centred log-ratio abundance across all samples (Mean CLR).

*******

| Primer | Sequence |
| --- | --- |
| Primary PCR- Forward Barcoding | 5’ ACACTCTTTCCCTACACGACGCTCTTCCGATCTNNNNNGTGCCAGCMGCCGCGGTAA 3’ |
| Primary PCR -  Reverse Barcoding | 5’ GTGACTGGAGTTCAGACGTGTGCTCTTCCGATCTGGACTACHVGGGTWTCTAAT 3’ |
| Secondary PCR – Forward Index | 5' AATGATACGGCGACCACCGAGATCTACAC[**8bpINDEX**]ACACTCTTTCCCTACACGACGCTC 3' |
| Secondary PCR – Reverse Index | 5' CAAGCAGAAGACGGCATACGAGAT[**8bpINDEX**]GTGACTGGAGTTCAGACGTGTGCTC 3' |
| Read 1 Sequencing | 5’ ACGTACGTACGGTGTGCCAGCMGCCGCGGTAA 3’ |
| Read 2 Sequencing | 5’ ACGTACGTACCCGGACTACHVGGGTWTCTAAT 3’ |
| Sequencing | 5’ ATTAGAWACCCBDGTAGTCCGGCTGACTGACT 3’ |

Table S1: Illumina amplification and sequencing primers used to assess bacterial microbiome communities from purified faecal DNA, taken from Caporaso et al, 2011


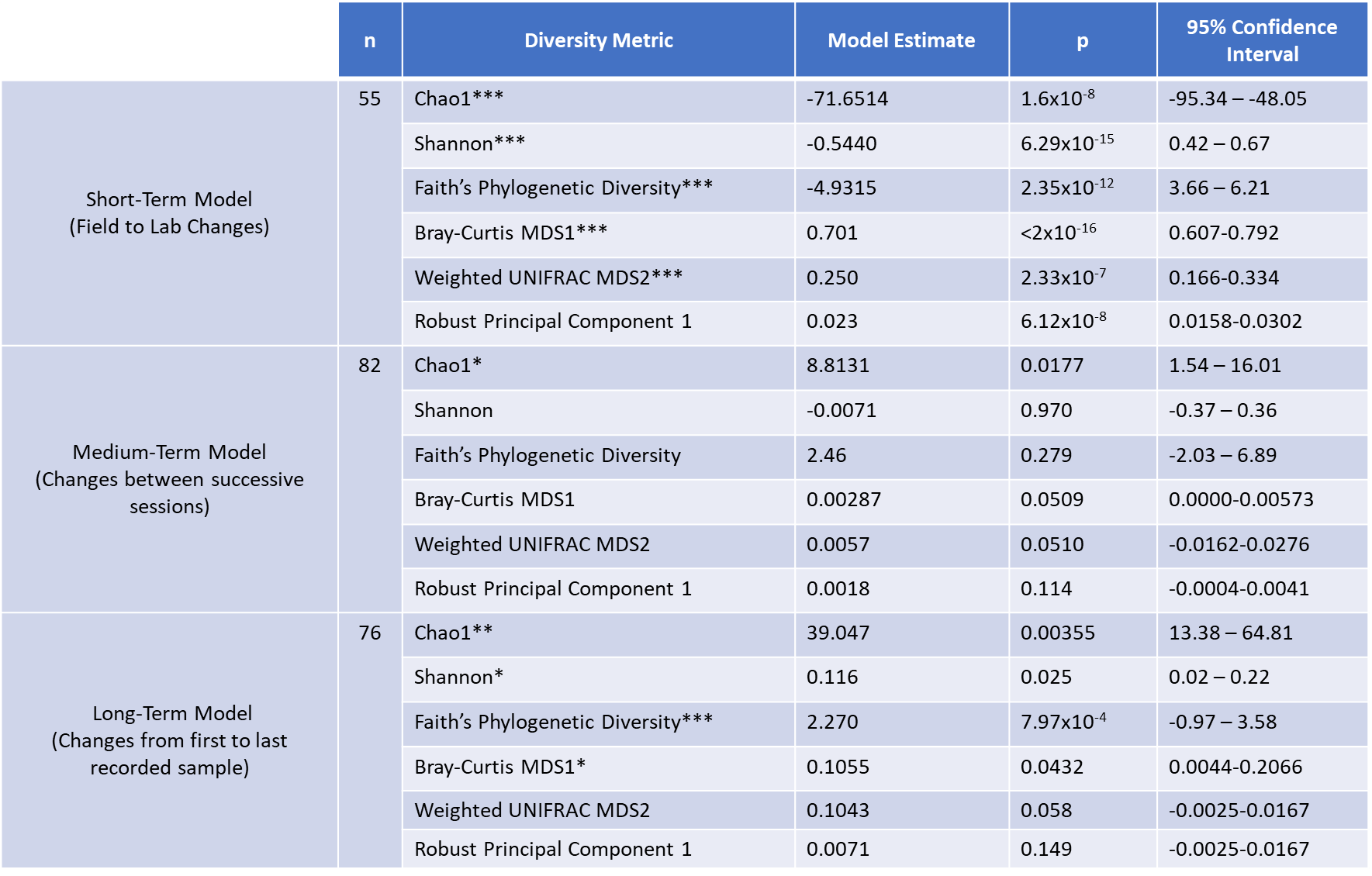


Table S2. Summary of model outputs from mixed-effects models showing changes in α-diversity (Chao1, Shannon Index & Faith’s Phylogenetic Diversity) and β-diversity (increasing Bacteroidetes dominance as represented by Bray-Curtis, Weighted UNIFRAC and RPCA site scores) associated with movement from the field to the laboratory over 1-2 days, across sequential trapping sessions, and from first to the last recorded capture per individual. Significance of associations are listed next to each metric (* p<0.05, ** p<0.01, *** p<0.001).


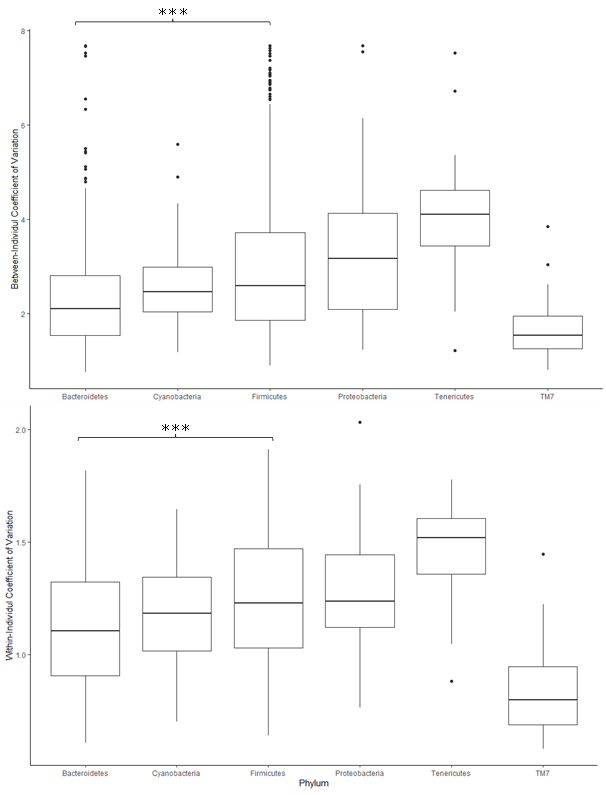


Fig S3. Differences across phyla in coefficient of variance in OTU relative abundance, as sampled from cross-sectional caecum and faeces microbiome sequencing data. Significant differences are shown between Firmicutes and Bacteroidetes (GLM with Tukey post-hoc: between individual p=<0.0001, within-individual p<0.0001), with Firmicutes showing a higher level of variance (* p<0.05, ** p<0.01, *** p<0.001).

Fig S4. Associations between ecological variables and microbiome diversity metrics. SMI (body condition) is positively associated with Chao1 (A) but not Shannon (B) α-diversity, negatively with Bray-Curtis MDS1 scores (C), but not wUniFrac MDS2 scores (D). Tapeworm infection is associated positively with Bracy-Curtis MDS1 scores (E), but not wUniFrac MDS2 scores (F) (* p<0.05, ** p<0.01, *** p<0.001).


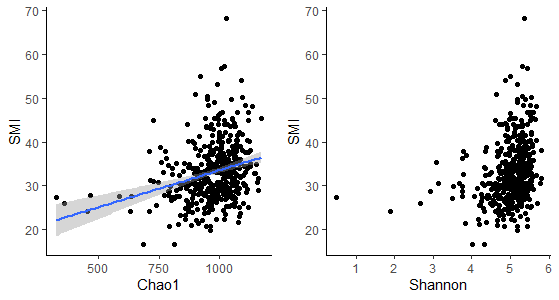

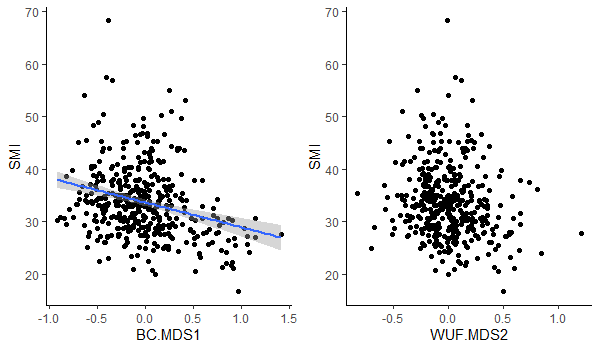

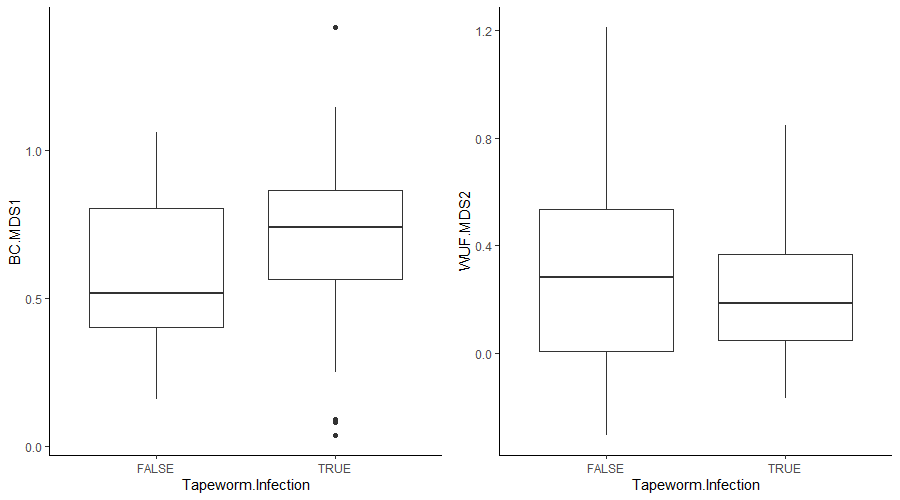


**A**

**B**

**C**

**D**

**E**

**F**

******

*****

*****

Table S3. OTUs showing strongest representation in Bray-Curtis MDS1 loading, with the 10 lowest and 10 highest loading values shown.


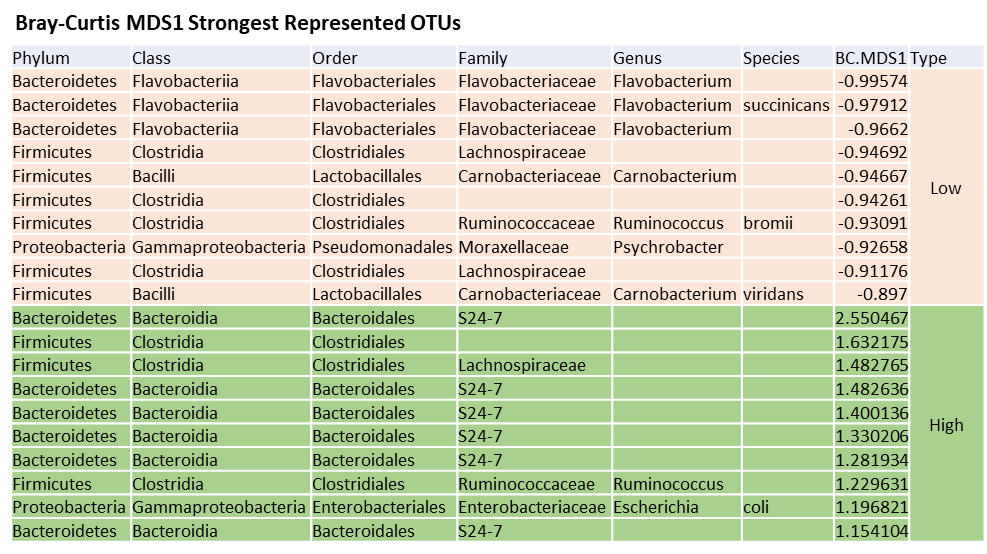


Table S4. OTUs showing strongest representation in wUniFrac MDS2 loading, with the 10 lowest and 10 highest loading values shown.


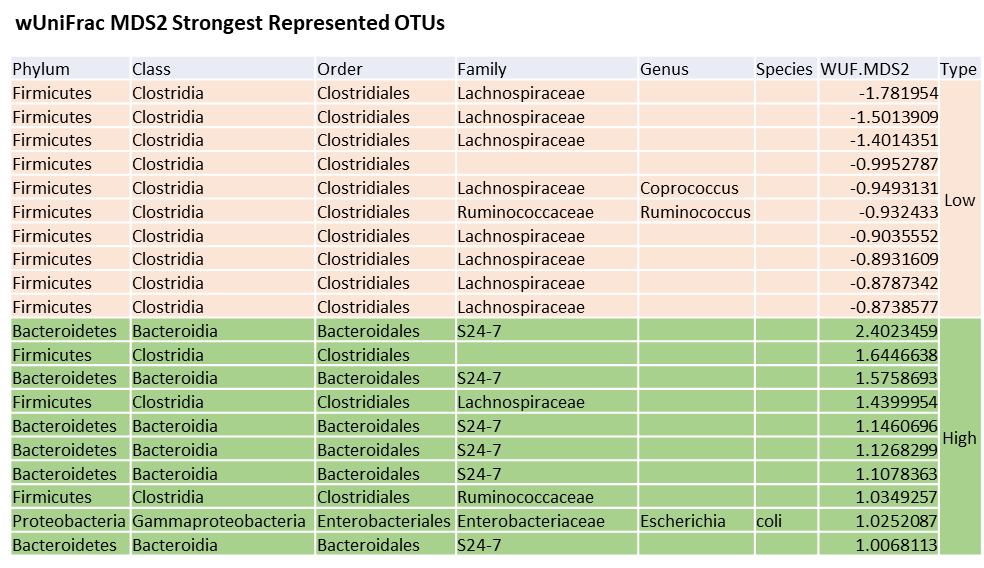


Table S5. OTUs showing strongest representation in Robust Principal component 1 loading, with the 10 lowest and 10 highest loading values shown.


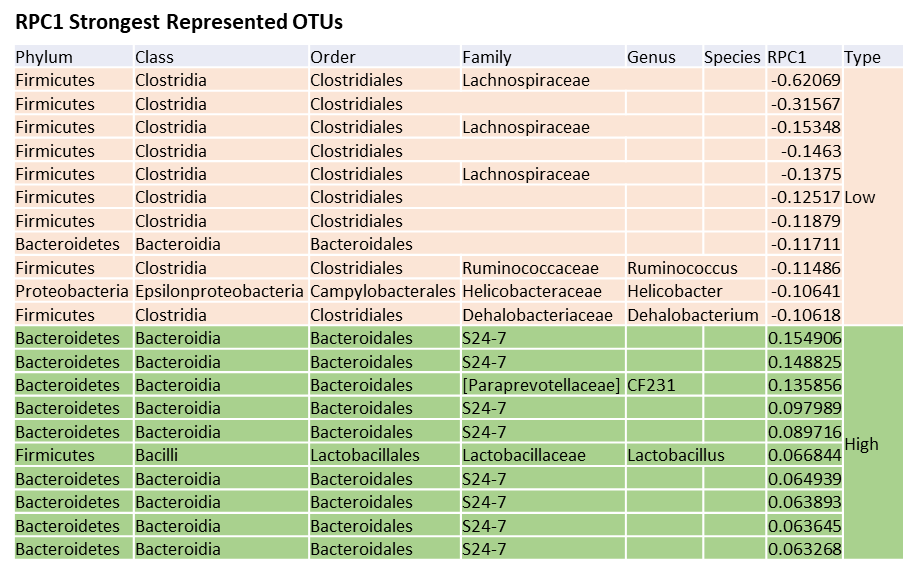

Supplement: Supplementary file 1 — Additional file 1. [file 12866_2023_2824_MOESM1_ESM.docx]
